# Early diagnostic value of serum procalcitonin levels for catheter-related blood stream infection in first-ever acute ischemic stroke patients

**DOI:** 10.1186/s12883-019-1557-2

**Published:** 2020-01-07

**Authors:** Yicheng Xu, Ruiwei Chen, Wei Qin, Peifu Wang, Peiyao Li, Wenli Hu, Jichen Du

**Affiliations:** 1 Department of Neurology, Aerospace Center Hospital, Beijing, 100049 China; 2 Department of Neurology, Peking university aerospace school of clinical Medicine, Yuquan Road ,NO.15,Haidian District, Beijing, 100049 People’s Republic of China; 3 Department of Neurology, Beijing Chaoyang Hospital, Capital Medical University, The Worker’s Stadium South Road, No. 8, Beijing, 100020 People’s Republic of China; 4 Department of Biomedical engineering, PLA general Hospital, Beijing, 100853 China

**Keywords:** Catheter-related infection, Diagnostic tests, Stroke, Procalcitonin

## Abstract

**Objective:**

The traditional approaches for diagnosing catheter-related bloodstream infection(CRBSI) is time consuming, which could not meet the clinical requirement. Our aim was to investigate the value of serum procalcitonin(PCT) in predicting CRBSI in first-ever acute ischemic stroke patients with central venous catheters (CVCs).

**Methods:**

This was a retrospective study. First-ever acute ischemic stroke patients hospitalized in neurological intensive care unit(NICU) of Aerospace Center Hospital and NICU of Beijing Chaoyang Hospital during January 2010 and December 2017 with clinically suspected CRBSI were enrolled. Peripheral blood white blood cell (WBC) count, neutrophils percentage(NE%), the levels of serum PCT, dwell time of catheterization and outcome of the patients were collected. According to the diagnosis of CRBSI or not, they were divided into CRBSI group and no CRBSI group. We used receiver operating characteristic curve (ROC) to evaluate the value of serum PCT levels in predicting CRBSI in patients with clinically suspected CRBSI.

**Results:**

Forty-five patients with suspected CRBSI were included in this study, and 13 patients were diagnosed with CRBSI. Comparing to those in no CRBSI group, the maximum body temperature (T_max_) (*p* = 0.036) and the PCT levels (*P* = 0.013) in CRBSI group were both significantly higher. The area under ROC of the serum PCT levels and the T_max_ to predict the CRBSI were 0.803 (0.95CI,0.660–0.946) and 0.680 (0.95CI,0.529–0.832) respectively. The PCT cut-off value was 0.780 ng/ml, with the sensitivity 69.23%, specificity 87.50%, positive predictive values 69.23% and negative predictive values 87.50%.

**Conclusion:**

It could be helpful to adopt PCT as a rapid diagnostic biomarker for first-ever acute stroke patients with suspected CRBSI.

## Background

Central venous catheters (CVCs) have become an important part in caring for severe patients, including those in neurological intensive care unit(NICU). However, as the adverse effect of CVCs usage, catheter-related bloodstream infection (CRBSI) could potentially increase the risk of mortality [1] and has become the main cause of health-care-associated infections [2]. The guidelines for the management of intravascular catheter-related infection (CRI) by the Infectious Diseases Society of America (IDSA) are still unclear about the strategy towards the patients with suspected CRBSI [3]. It is difficult for physicians working in the ICU to deal with the situation of patients with CVCs and suspected CRBSI.

Following reasons can be put forward to explain the difficulty. First, the traditional approaches require the confirmation of CRBSI which was based on the culture of catheter tips and peripheral blood. And it was also time consuming. Meanwhile it often needs to remove catheters and initiate early empirical antibiotic therapy [3, 4]. Second, one important reason for physicians to suspect CRBSI and then to remove the central venous catheter was the presence of fever. However, it had been found that not only the infectious etiologies but also a great number of non-infectious etiologies were responsible for the unexplained fever of patients in ICU [5], especially in NICU. Studies had found that up to 70% or more catheters removed for suspected CRBSI were proved to be unnecessary, which also resulted in increased risk of iatrogenic complications, unnecessary antibiotics use and increased health care expenditures [6, 7].

Hence it is important to develop a nonculture-based diagnostic method to offer useful adjuncts to conventional approaches. As a sepsis-induced protein, serum procalcitonin(PCT) is ubiquitously released in response to bacterial toxins and other pro-inflammatory cytokines (e.g., interleukin-1β, interleukin-2, interleukin6 and tumor necrosis factor-α) and are also downregulated when the concentrations of these substances decrease as patient’s recovery from infection [8, 9]. Consequently studies have found that PCT have good diagnostic value for differentiating serious bacterial infections from nonbacterial infections [10]. Furthermore, low serum PCT levels have an accurate ability to rule out the diagnosis of bacteremia in patients with acute fever [11], which may become a useful test to avoid unnecessary removal of catheters and unnecessary antibiotics usage in suspected CRBSI patients. Therefor our aim was to investigate the value of serum procalcitonin(PCT) in predicting CRBSI in first-ever acute ischemic stroke patients with central venous catheters (CVCs).

## Methods

First-ever acute ischemic stroke patients hospitalized in NICU of Aerospace Center Hospital and NICU of Beijing Chaoyang Hospital, Capital Medical University between January 2010 and December 2017 with clinically suspected CRBSI and eligible for inclusion were enrolled. Patients who have reached 18 years of age or older got first-ever acute ischemic stroke which defined in accordance with the World Health Organization criteria [12], and accepted CVC for treatment during NICU were eligible for inclusion. The institutional review board of our hospital approved this retrospective study; Patient informed consent for inclusion in this study was waived.

Exclusion criteria of the study were as follows: (1) age less than 18 years; (2) patients who had an indwelling CVC before admitted to NICU; (3) patients who had fever within 48 h after accepted CVC. (4) patients with autoimmune diseases or tumor; (5) neutropenic patients (< 500/mm^3^); (6) patients with incomplete data; (7) patients who early discharged. Clinical suspicion of CRBSI was considered if patient had the following symptoms simultaneously: (1) clinical manifestations of acute infection (fever, chills, and/or hypotension); (2) indwelling CVC for over 48 h; (3) no other obvious source of infection [3, 13]. Or the physicians judged that the above clinical manifestation of acute infection could not be well explained by the current source of infection and for most cases, the physicians usually decided to remove the CVC immediately.

On the basis of previously published guidelines [3], CRBSI could be definitely diagnosed when they met the following conditions: 1.the organism isolated from the catheter tip culture and one peripheral blood culture were same, or 2. positive culture obtained from both blood from the peripheral vein and the catheter hub, meeting the CRBSI criteria for differential time to positivity (DTTP) [4, 14].

For all patients, clinical and demographical data, including: age,sex, history of diabetes, smoking history, hypertension, location of cerebral infarction, and National Institute of Health stroke scale (NIHSS) scores at baseline were collected. Clinical and laboratory data in NICU including maximum body temperature (T_max_) of patients before suspected CRBSI, peripheral white blood cell count (WBC), neutrophil percentage(NE%), serum PCT levels, time of dwelling catheter and patients outcome in NICU were also collected.

### Statistical analysis

SPSS software (version 20; SPSS Inc., Chicago, IL, USA) was used for data analysis.

Parametric values were presented as mean(M) ± standard deviation (SD), nonparametric values were presented as a median [interquartile ranges (IQRs)]. Comparison between the two groups was examined by using Student *t* test or the Mann-Whitney *U* test after testing for normality. To analyse the categorial variables, the *χ2* test or Fisher exact test was adopted. Receiver operating characteristic (ROC) curve was performed to determine the optimal cutoff value of the serum PCT levels. The area under the ROC curve(AUC), sensitivity, specificity, positive predictive values and negative predictive values were calculated. In all analyses, A *p*-values < 0.05 was regarded as statistically significant.

## Results

Clinical suspicion of CRBSI occurred in 45 patients (31.1% female, *n* = 14; 68.9% male, *n* = 35). We listed the demographic and clinical features of the patients in Table 1. The mean age was 73.8 ± 13.1. Anterior circulation infarction occurred in 13 cases, posterior circulation infarction occurred in 21 cases and 11 cases for both. Mean NIHSS score on admission was 14.9 ± 6. According to the diagnostic criteria of CRBSI, 13 (28.9%) of these 45 patients, were positive for CRBSI and the remaining 32(71.1%) were negative for CRBSI. There were 4 patients (30.7%) in CRBSI group given antibiotic before bacterial culture, and 14 patients (43.8%) in no CRBSI group given antibiotic before bacterial culture (*P* = 0.42). PCT levels, maximum body temperature (T_max_) and NICU mortality were obviously lower in no CRBSI group than that of CRBSI group(*P* < 0.05). No statistically significant differences of age, gender, NIHSS score and WBC count were observed between CRBSI group and no CRBSI group. The microbes causing CRBSI were methicillin-resistant Staphylococcus aureus(*n* = 1), *Candida glabrata* (*n* = 1), *Klebsiella pneumoniae* (*n* = 1), multiresistant Acinetobacter baumannii (*n* = 2), *Pseudomonas aeruginosa* (*n* = 2) and coagulase-negative staphylocci (*n* = 6).

**Table 1:** Demographic features and laboratory findings between two group

	CRBSI(*n* = 13)	No CRBSI (*n* = 32)	*T /χ2*	*P*
Age (years)	71.1 ± 16.7	74.9 ± 11.4	−0.881	0.383
Sex (male/female)			0.150	0.699
Male	10 (23.1)	21(65.6)		
Female	3 (76.9)	11(34.4)		
NIHSS scores	13.8 ± 7.1	15.4 ± 5.6	−0.770	0.446
History of diabetes	7	14	0.379	0.538
Smoking history	10	23	0.120	0.729
Hypertension	6	16	0.055	0.815
Location of cerebral infarction			0.838	0.658
Anterior circulation	4(30.8)	9 (23.1)		
Posterior circulation	7(53.8)	14(43.8)		
Both	2(15.4)	9 (23.1)		
Previous antibiotic therapy no.(%)patients	4(30.7)	14(43.8)	0.649	0.420
Position			3.740	0.154
subclavian	6(46.1)	6(18.8)		
jugular	5(38.5)	16(50)		
PICC	2(15.4)	10(31.3)		
Tmax (°C)	38.65 ± 0.57	38.12 ± 1.06	2.167	0.036
WBC (× 10^9^/L)	13.85 ± 7.1	15.38 ± 5.6	1.198	0.246
NE%	79.3 ± 8.1	80.1 ± 7.1	−0.328	0.744
PCT (ng/ml)	2.783 ± 2.451	0.756 ± 1.250	2.835	0.013
Time of dwelling	20.0(11.0,30.0)	17(10.5,22.75)	0.535	0.465
catheter(M(1q,3q))				
Motality during NICU	7/13	5/32	5.090	0.024

Note: the NIHSS score is the National Institutes of Health Stroke Scale, PICC is peripherally inserted central catheter and Tmax is the highest body temperature

**Fig. 1:**
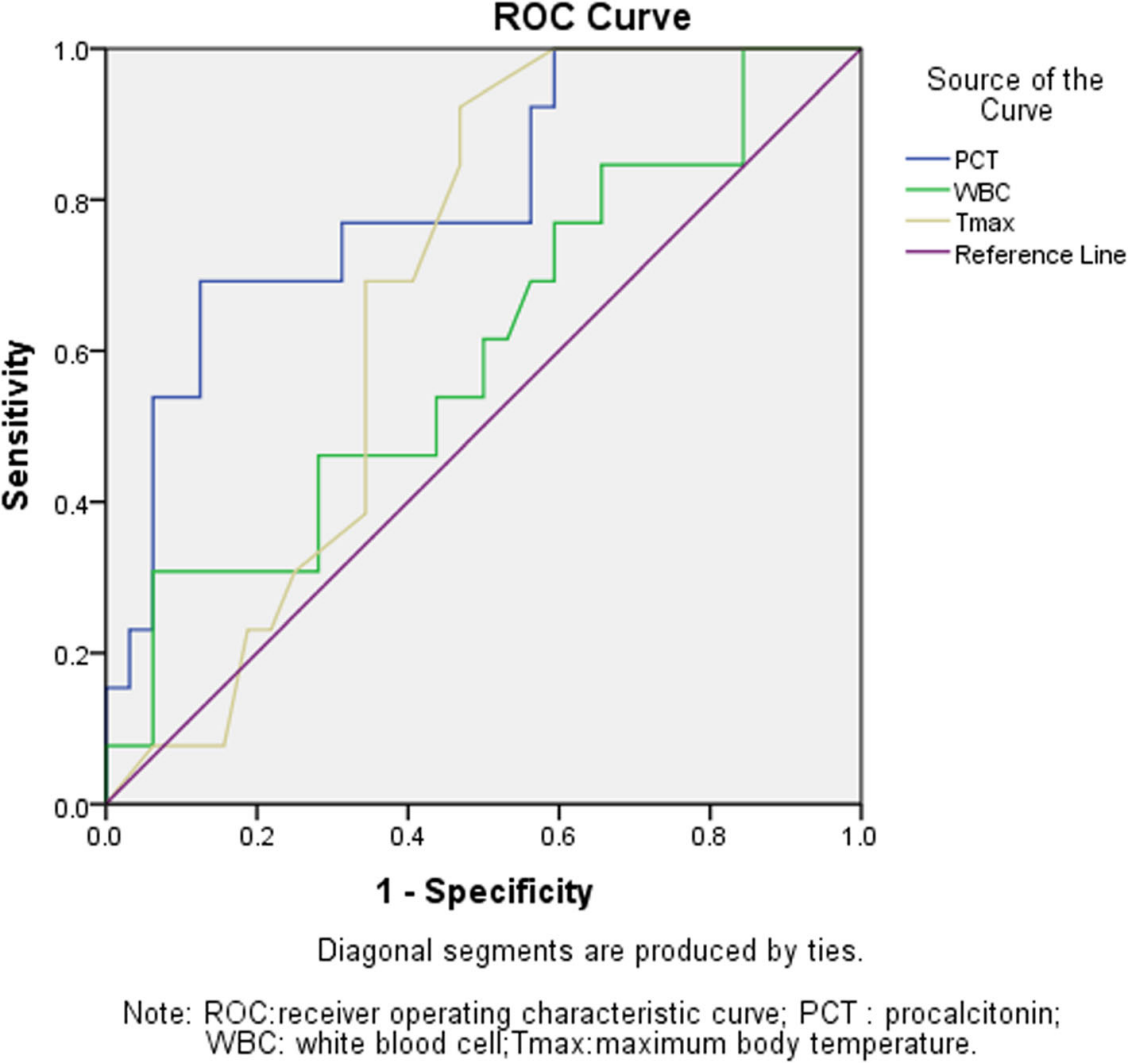
Receiver operating characteristic curves for procalcitonin(PCT), maximum body temperature (T_max_) and white blood cell count (WBC) for the prediction of catheter-related bloodstream infection (CRBSI)

The AUC for the serum PCT levels, T_max_ and WBC in CRBSI prediction were 0.803 (95% CI, 0.660–0.946; *P* = 0.002), 0.680 (0.95%CI, 0.529–0.832; *P* = 0.060) and 0.602 (95% CI, 0.417–0.787; *P* = 0.287)respectively. The optimal cutoff value for serum PCT levels to predict CRBSI was 0.78 ng/ml, with the sensitivity 69.23%, specificity 87.50%, positive predictive values 69.23% and negative predictive values 87.50% (Fig. 1). Meanwhile, the PCT cut off value of 0.32 ng/ml showed the highest Sensitivity(92.31%) and highest negative predictive value (92.9%) (Table 2).

**Table 2:** Sensitivity(Se), specificity(Sp),positive predictive value (PPV) and negative predictive value (NPV) of PCT in predicting CRBSI

PCT(cut-off value)ng/ml	Se%	Sp%	PPV%	NPV%
0.28	100	37.5	39.4	100
0.32	92.31	40.63	38.7	92.9
0.52	76.92	68.75	50	88
0.78	69.23	87.5	69.2	87.5

## Discussion

In our study, we investigated the early diagnostic ability of serum PCT levels for CRBSI in patients with first-ever acute ischemic stroke hospitalized in NICU. We found that the level of PCT provide more prediction accuracy of CRBSI compared with T_max_, WBC count and neutrophils percentage in patients with first-ever acute ischemic stroke. Furthermore, on the day when CRBSI was suspected, serum PCT levels > 0.780 ng/ml, were excellent with respect to distinguish between patients with CRBSI and patients without. The PCT cut off value of 0.32 ng/ml had the highest Sensitivity and highest negative predictive value.

It has been well recognized that CRBSI has association with increased risk of mortality [1, 15]. And our result was in line with these findings(53.8% VS 15.6%). However, as current clinical guidelines for intravascular catheter-related infection management from the Infectious Diseases Society of America (IDSA) are still ambiguous on the treatment strategy for the patients with suspected CRBSI [3], Controversy still exists about the correct timing to remove the catheter when CRBSI is suspected. Studies had reported that a large proportion of catheters removal for suspected CRBSI were proved to be unnecessary, and it can be up to 70% or more [16, 17]. Consistent with the above findings, our result found that 65% of the CVC removal in our study were proved to be sterile. Our study also found that the PCT cut off value of 0.32 ng/ml showed the highest Sensitivity and highest negative predictive value. Which means low PCT levels (≤0.32 ng/ml) had good predict value of absence of CBRSI in first-ever acute ischemic stroke patients. This point might be adopted by physicians to avoid unnecessary removal of catheters and unnecessary antibiotics usage in suspected CRBSI patients with ischemic stroke in the real clinical setting.

Several studies have evaluate the PCT’s predictive value for different infections in the ICU setting [18–20]. However, only two studies have investigated the early diagnostic value of serum PCT levels for CRBSI among different adult patient cohort, not included patients in NICU. In a prospective observational study included 46 patients with suspected CRBIS infection, the authors advised that PCT could have a useful diagnostic ability of CRBSI in medico-surgical ICU setting [4]. Another study found that in patients after orthotopic liver transplantation, PCT also has a useful rapid diagnostic ability for the detection of CRBIS in those patients [21]. Consistent with these findings, our study indicated that serum PCT levels were helpful in the early diagnosis of CRBSI in first-ever stroke patients.

Our study has some limitations. First, the sample sizes were relatively small. Larger population sample studies are expected to validate our results. Second, in current study, there were 30.7% patients in CRBSI group and 43.8% patients in no CRBSI group administered antibiotic before bacterial culture (*P* = 0.42). Which might have resulted in negative bacterial culture and false negative of CRBSI. However it was the real and common situations that physicians in NICU had to deal with. We thought it might be considered as the meaningful point of our study for real clinical settings. Third, only first-ever acute ischemic stroke patients were included. Therefore it should be cautious to extrapolate our results to other disease conditions. Fourth, as the sample population was small, we failed to investigate the prognostic ability of serum PCT levels in CRBSI patients.

## Conclusion

It could be helpful to adopt PCT as a rapid diagnostic biomarker for first-ever acute stroke patients with suspected CRBSI. Further study with larger cohort size is required to validate our findings and evaluate the effectiveness of PCT levels in preventing unnecessary removal of CVCs and antibiotic overuse.

## Data Availability

The data that support the findings of this study are available from the corresponding author upon reasonable request.

## Abbreviations

CVCs: central venous catheters
NICU: neurological intensive care unit
CRBSI: catheter-related bloodstream infection
CRI: catheter-related infection
IDSA: Infectious Diseases Society of America
PCT: procalcitonin
DTTP: differential time to positivity
NIHSS: National Institute of Health stroke scale
Tmax: maximum body temperature
WBC: white blood cell count
NE%: neutrophil percentage
ROC curve: Receiver Operating Characteristic curve
AUC: area under the ROC curve
